# Force–velocity relationship in Paralympic powerlifting: two or multiple-point methods to determine a maximum repetition

**DOI:** 10.1186/s13102-022-00552-9

**Published:** 2022-08-24

**Authors:** Felipe J. Aidar, Ciro José Brito, Dihogo Gama de Matos, Levy Anthony S. de Oliveira, Rapahel Fabrício de Souza, Paulo Francisco de Almeida-Neto, Breno Guilherme de Araújo Tinoco Cabral, Henrique P. Neiva, Frederico Ribeiro Neto, Victor Machado Reis, Daniel A. Marinho, Mário C. Marques, Filipe Manuel Clemente, Hadi Nobari

**Affiliations:** 1 Department of Physical Education, Federal University of Sergipe (UFS), São Cristovão, 49100-000 Brazil; 2 Group of Studies and Research of Performance, Sport, Health and Paralympic Sports (GEPEPS), Federal University of Sergipe (UFS), São Cristovão, 49100-000 Brazil; 3 Graduate Program of Physiological Science, Federal University of Sergipe (UFS), São Cristovão, 49100-000 Brazil; 4 Graduate Program of Physical Education, Federal University of Sergipe (UFS), São Cristovão, 49100-000 Brazil; 5 Graduate Program in Physical Education, School of Physical Education and Sport, Federal University of Juiz de Fora, Governador Valadares, 36036-330 Brazil; 6 Cardiorespiratory and Physiology of Exercise Research Laboratory, Faculty of Kinesiology and Recreation Management, University of Manitoba, Winnipeg, MB Canada; 7 Health Sciences Center, CCS-UFRN, Federal University of Rio Grande Do Norte, Natal, RN 59012-570 Brazil; 8 Department of Sport Sciences, Research Center in Sports Sciences, Health Sciences and Human Development (CIDESD), University of Beira Interior, 6201-001 Covilhã, Portugal; 9 Paralympic Sports Program, SARAH Rehabilitation Hospital Network, Brasilia, 71535-005 Brazil; 10 Research Center in Sports Sciences, Health Sciences and Human Development (CIDESD), University of Trás-Os-Montes E Alto Douro, 5001-801 Vila Real, Portugal; 11 Escola Superior Desporto E Lazer, Rua Escola Industrial E Comercial de Nun’Álvares, Instituto Politécnico de Viana Do Castelo, 4900-347 Viana do Castelo, Portugal; 12 Delegação da Covilhã, Instituto de Telecomunicações, 1049-001 Lisbon, Portugal; 13 Department of Exercise Physiology, Faculty of Educational Sciences and Psychology, University of Mohaghegh Ardabili, Ardabil, 56199-11367 Iran; 14 Department of Physical Education and Special Motricity, Faculty of Physical Education and Mountain Sports, Transilvania University of Braşov, 500068 Braşov, Romania; 15 Faculty of Sport Sciences, University of Extremadura, Avenida de La Universidad, 10003 Cáceres, Spain

**Keywords:** Paralympics, Powerlifting, Force–velocity relationship, Multiple-load method, Load estimation

## Abstract

**Background:**

Due to the absence of evidence in the literature on Paralympic Powerlifting the present study investigated various methods to assess bench press maximum repetition and the way each method influences the measurement of minimum velocity limit (MVT), load at zero velocity (LD0), and force–velocity (FV).

**Objective:**

To evaluate the precision of the multi-point method using proximal loads (40, 50, 60, 70, 80, and 90% of one repetition maximum; 1RM) compared to the four-point method (50, 60, 70, and 80% of 1RM) and the two-point method using distant loads (40 and 80% and 50 and 80% of 1RM) in in the MVT, LD0, and FV, in bench press performed by Paralympic Powerlifters (PP).

**Methods:**

To accomplish this, 15 male elite PP athletes participated in the study (age: 27.7 ± 5.7 years; BM: 74.0 ± 19.5 kg). All participants performed an adapted bench press test (free weight) with 6 loads (40, 50, 60, 70, 80, and 90% 1RM), 4 loads (50, 60, 70, and 80% 1RM), and 2 loads (40–80% and 50–80% 1RM). The 1RM predictions were made by MVT, LD0, and FV.

**Results:**

The main results indicated that the multiple (4 and 6) pointsmethod provides good results in the MVT (R^2^ = 0.482), the LD0 (R^2^ = 0.614), and the FV (R^2^ = 0.508). The two-point method (50–80%) showed a higher mean in MVT [1268.2 ± 502.0 N; ICC95% 0.76 (0.31–0.92)], in LD0 [1504.1 ± 597.3 N; 0.63 (0.17–0.86)], and in FV [1479.2 ± 636.0 N; 0.60 (0.10–0.86)].

**Conclusion:**

The multiple-point method (4 and 6 points) and the two-point method (40–80%) using the MVT, LD0, and FV all showed a good ability to predict bench press 1RM in PP.

## Background

Much has been studied about the influence of physical activities on health and psychosocial aspects, mainly in people with some type of pathology or disability, or from special groups [1–7]. On the other hand, the practice of physical activities and sports are important for people with disabilities [8, 9]. Regarding Paralympic sports, Paralympic Powerlifting (PP), an adapted sport [10], has been gaining more supporters worldwide [11, 12] and presents various adaptations to training, especially concerning mechanical variables [13]. PP athletes tend to develop great strength, though with low training and competition velocities or in differentiated regimes [14–16]. Among the mechanical variables herein, the relation of force–velocity (F–V) tends to be very important and presents a linear relationship. It has been used to evaluate the capacities in the maximum production of force (F0), velocity (V0), and power (Pmax) in diverse activities and bench press [17, 18]. Thus, studies have revealed that the results of the F–V relationship (F0, V0, F–V slope, and Pmax) can be used to implement individualized programs [19–21]. However, a basic prerequisite for the adequacy of training programs based on the FV profile is that the main results must be highly reliable.

The test procedure in relation to FV during iso-inertial tasks, consists in the application of multiple (at least four) loads (Multiple-Point Method—MPM) [18, 22]. Studies carried out with the MPM showed a highly linear relationship to F–V, with high reliability and moderate to high validity [18, 23–26]. However, due to the high linearity of the relationship with FV, Jaric [27] proposed a test based on the application of only two loads (2PM). Studies have confirmed the similar reliability and high validity of the relation to F–V obtained from MPM and 2PM [17, 25–31]. However, it has been shown that the accuracy of the two-point method (2PM) depends on several factors such as the distance between the two experimental points, the proximity of the points to the F and V intercepts and the reliability of the individual points [18, 28, 32, 33]. Furthermore, it was found that the reliability and validity of the two-point method progressively decreased as the separation between the two experimental points was reduced [27, 28].

The relationship of F–V to MPM has been widely applied in the control of strength training loads, especially when related to load-velocity [33, 34]. However, regarding PP, studies have focused more on the health issues related to the etiology of injuries [8, 35]. In PP, the extended legs on the bench tend to reduce the transfer of force to lifting [36], in which affects the maintenance of force, power, velocity, and, and these are not well elucidated [16, 37, 38].

A practical question that remains open is whether there is accuracy of the F–V relation in disabled athletes performed on the adapted bench press [14, 16, 18, 39] and whether this affected by its different methods of evaluation. Another point that does not present consensus so far is which of the three evaluation methods would be most effective for calculating force and velocity indicators, namely the minimum velocity limit (MVT), zero velocity load (LD0), and force–velocity (FV) [40, 41]. Therefore, the aim of the present study was to evaluate the precision of the multi-point method using proximal loads (40, 50, 60, 70, 80, and 90% of one repetition maximum; 1RM) compared to the four-point method (50, 60, 70, and 80% of 1RM) and the two-point method using distant loads (40 and 80% and 50 and 80% of 1RM) in the MVT, LD0, and FV, in bench press performed by Paralympic Powerlifters (PP).Our hypothesis was that (i) the multiple (4 and 6) points method provides a good relationship with FV in the adapted bench press for disabled people and national-level Paralympic Powerlifting athletes; (ii) the MVT, LD0, and FV methods present reliable values at the evaluated points and; (iii) the two-point methods can be used with good reliability and (iv) the use of free weights presents good reliability in the adapted bench press for disabled people and PP national level athletes.

## Method

### Experimental approach to the problem

**Fig. 1:**
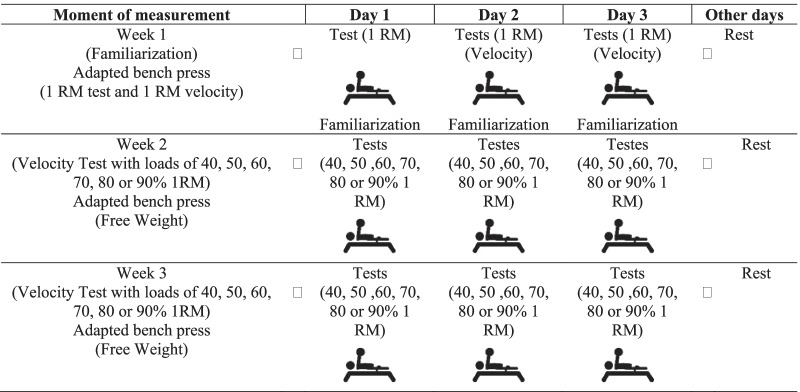
Experimental approach. RM: Repetition Maximum

Subjects were laboratory tested nine times over 21 days (3 weeks) and each session was separated by at least 24 h. First week: The purpose of the first visit was to familiarize the subjects with the Adapted Bench Press test protocol and the desired technique for the tests, before determining the Adapted Bench Press 1RM and minimum velocity limit (i.e., 1RM velocity). On the second and third visits, participants completed a protocol where they progressively lifted heavier loads (40 to 90% 1RM), during which concentric velocity was monitored to establish individual velocity and load relationships. In these visits, the 1RM of the Adapted Bench Press and the minimum velocity limit (i.e., 1RM velocity) were determined. In weeks "2" and "3", the subjects were tested with loads of 40, 50, 60, 70, 80, and 90% of 1RM, performed in six sessions with loads defined randomly (by drawing lots) with a minimum rest interval of 24 h (resulting in three sessions per week). The 1RM predictions were made by linear regression with the use of the minimum velocity limit (MVT), the load at zero velocity (LD0) or the force–velocity (FV) as predictors [41], using the load-velocity relationships developed using six loads (40, 50, 60, 70, 80, and 90% 1RM), four loads (50, 60, 70, 80% 1RM) and two loads (40–80% and 50–80% 1RM). This experimental approach allowed the study of the influence of performing exercises using free weights (Adapted Bench Press) on the reliability and validity of using load-velocity relationships to predict 1RM. Each participant performed all sessions at the same time of the day and under similar environmental conditions (22–25 °C). Figure 1 shows the experimental design of the study.

### Participants

15 male elite PP athletes (age: 27.7 ± 5.7 years; experience: 2.1 ± 0.9 years; body mass: 74.0 ± 19.5 kg; 1RM: 113.0 ± 31.3 kg; 1RM/BM: 1.6 ± 0.3) were included, all participating in an training program at the university where the study was conducted. It was required that all participants were nationally classified competitors, eligible to compete in the adapted sport, had participated in at least one competition at the national level during the previous year, and had a nine-month minimum experience in the sport. Among the participants, six athletes presented spinal cord injury due to accidents with injuries below the eighth thoracic vertebra; four amputees, two with polio, one with Cerebral Palsy, and two with *Arthrogryposis.* The participants abstained from strenuous exercise for a minimum period of 48 h before each test session and were instructed not to consume alcohol and coffee during this period. All participants who started the study performed all tests and there were no exclusions.

The participants were evaluated during the competitive phase of the season and were familiar with the testing procedures due to constant training and testing routines. All participants signed a free and informed consent term. This study was conducted under the ethical principles set out in the Helsinki Declaration (2013). It was approved by the Research Ethics Committee of the Federal University of Sergipe, CAAE: 2.637.882 (date of approval: 7 May 2018).

### Procedures

During the first visit, for familiarization purposes, body mass was measured while sitting on a Micheletti Electronic^®^ Wheelchair Scale (Micheletti^®^, São Paulo, Brazil) with a maximum weight capacity of 300 kg (dimensions of 5.0 × 102 × 120 cm). Then, the 1RM load and the velocity limit for 1RM were assessed. In the following sessions, each participant completed a progressive load protocol to establish the specific load and velocity relationships. To perform the bench press, an official straight bench (Eleiko Sport AB^®^, Halmstad, Sweden) approved by the International Paralympic Committee was chosen, with a total length of 210 cm. The bar used was the 220 cm Eleiko brand (Eleiko Sport AB^®^, Halmstad, Sweden), weighing 20 kg.

### Warm-up

Participants performed a pre-warm-up for the upper limbs that consisted of three exercises (shoulder abduction with dumbbells, development of the shoulders in the machine, rotation of the shoulders with dumbbells), with a series of 20 repetitions for approximately 10 min. Then, a specific warm-up was performed on the bench press using only the bar (20 kg) without extra weight, completing10 slow repetitions (3.0 × 1.0 s, eccentric × concentric) and 10 fast repetitions (1.0 × 1.0 s, eccentric × concentric). Then, the subjects performed five repetitions with 40% of 1RM, followed by three repetitions with 50% of 1RM, one repetition with 70, one with 80, and one with 90% of 1RM. Between sets, participants rested for at least three minutes [37, 38].

### Load Determination

The participants started the tests with a self-selected load estimated to be the maximum load. Weight was then added until the maximum load was attained. If the participant overestimated the initial load, 2.5% of the load was subtracted before a new attempt [42]. A rest of 3.0 to 5.0 min was provided between trials, according to the participants' perception of recovery [42–44]. The coefficient of variation between the two measures was at least 94%.

To determine the test load, the 1RM load was assessed. Each participant started the attempts with a weight that could only be lifted once with maximum effort and selected the grip width that was most comfortable for each participant. The VMP in 1RM was 0.17 m/s, a value used as a reference value for the MVT method.

The determination of 1RM was performed in the first week [16, 41]. To determine the predicted values of 1RM performed in the second and third weeks, the velocity at each load was evaluated using 3 repetitions at 40% of the predicted 1RM (estimated by the subject), 3 repetitions at 50% 1RM, 3 repetitions at 60% 1RM, 3 repetitions at 70% 1RM, 1 rep at 80% 1RM, and 1 rep at 90% 1RM [45, 46], with 3 min of rest between the velocity tests. In the case of 70, 80, and 90%, three tests of one repetition were performed, so that the velocity of the three repetitions was obtained.

During all tests, participants were instructed to perform the adapted bench press exercise with the eccentric phase performed under control, while the concentric phase was completed as quickly as possible. With the bar (Eleiko Sport AB^®^, Halmstad, Sweden) supported with the elbows extended, the participant flexed the elbows until the bar touched the chest. The position close to the nipple lines was demarcated with a towel to keep the movement as linear as possible, signaling the most appropriate path of the bar, which was visually confirmed by the researchers positioned adjacent to the participant. Participants were also given verbal cues about when to stop the eccentric phase (approximated stop and 1.0 s) and start the concentric phase of the bench press, along with consistent verbal encouragement [41].

During each lift, the displacement of the bar and the time between data points were recorded using a linear position transducer sampling cable up to 50 Hz (Force Measurement System Speed4Lift SL^®^; Mostoles, Madrid, Spain) [47]. From these data, the concentric phase of each repetition was automatically identified by the linear position transducer and the average concentric velocity was calculated for that part of the lift. This linear position transducer was previously validated [41].

The retractable cord of this device was fixed inside the bar collar, with the unit mounted on the floor directly below the position of the bar during the bench press action. The use of the fastest repetition in each load ensured that the velocities used to develop the load-velocity relationship represented the individual's best performance. With lighter loads (< 80% 1RM), the fastest repetition tends to be the second or third, especially in warm-up sets. Subsequent analysis was performed to predict 1RM from these load velocity profiles using three different methods: (1) MVT; (2) LD0 and; (3) FV (Fig. 1).

### Load velocity relations

The average concentric velocity and concentric force of each repetition were quantified using a previously validated linear position transducer (Force Measurement System Speed4Lift SL^®^; Mostoles, Madrid, Spain) [47]. Individual load-velocity relationships specific to each exercise were developed for each participant. These relationships only included the highest velocity repetition measured in the tests (40–90% 1RM).

Subsequent analyses were performed to predict 1RM from the load-velocity relationship using three different methods applied previously: (1) MVT; (2) LD0 and; (3) FV. The specific calculations used to determine the predicted 1RM for each of these methods were based on previous studies [41, 45]. These 1RM prediction calculations were performed using the full load-velocity relationships developed using 6 loads (40%, 50%, 60%, 70%, 80%, and 90% 1RM), with 4 loads (50%, 60%, 70%, and 80% of 1RM), and in two 2-load methods (40% and 80%; 50% and 80%) that tend to be more viable due to the exclusion of heavier loads [16].

The regression equation to estimate 1RM was done using three different predictors: MVT, LD0 and FV [41], and for each participant it was taken into account the load and velocity values at every relative load (40–90% 1RM). The MVT method was based on the assumption that in 1RM the failure will always occur at the same velocity (for the same participant and exercise) [41]; commonly referred to as the MVT. After identifying the MVT, the regression to the specific load-velocity ratio equation was determined for the MVT to predict 1RM (1RMMVT) [40], which is also suitable for the prediction of 1RM in the bench press with free weight. The LD0 method presents a load-velocity relationship and the regression would occur at a velocity of 0.0 m.s^−1^ [39], performed with free weight, and the mean estimates of 1RMLD0 of free weight did not differ from the measured 1RM [41]. The FV method relates the force with the velocity, and subsequently, the determination of the individual FV 1RM intercept and the load-velocity relationship, above the gravity acceleration (9.81 m.s^−2^) [22].

### Statistical Analysis

Measures of mean central tendency ± Standard Deviation (X ± SD) were used. The test reliability of the predicted scores of 1RMMVT, 1RMLD0, and 1RMFV was determined by comparing the 1RM predictions between tests. The intraclass correlation coefficient (ICC: 3.1) and the coefficient of variation (CV) with 95% confidence intervals were calculated using a custom spreadsheet developed for this purpose [48]. These form values are used to calculate the ICC and CV values for individual points in the load-velocity relationship in the experiments to examine reliability. The ICCs were classified according to the following criteria: excellent (ICC = 0.91–1.00), good (ICC = 0.76–0.90), moderate (ICC = 0.51–0.75), and poor (ICC = 0.00–0.50). The magnitude of the CV was based on the following parameters: bad (> 0.10%), moderate (5–10%) and good (5%). Pearson's product-moment correlation coefficients were calculated to assess the relationships between measured and predicted 1RM scores. Bland–Altman plots were used to describe the level of agreement between measured and predicted 1RM values and identify significant trends in the Bland–Altman plots [49]. Pearson correlation analysis was used to examine the relationship between the variables "x" (mean score of 1RM) and "y" (difference between predicted and measured 1RM) of each graph to identify any trends in the data. The one-way ANOVA with repeated measures was performed to assess differences in measured versus predicted scores of 1RM during trials and Bonferroni Post Hoc. In cases where sphericity was violated, the Greenhouse–Geisser correction procedure was used. In circumstances where a significant main effect was observed, post hoc Fisher’s Least Significant Differences analyses were performed to determine where these differences occurred [50, 51]. These analyses assessed whether the 1RMMVT, 1RMLD0, and 1RMFV predictions were reliable and could be used to accurately determine 1RM. Significance was established with a type I error rate of α ≤ 0.05, and these analyses were performed using SPSS (v.25, IBM, New York, USA) and Prisma GraphPad version 8.1 (GraphPad Software, San Diego, California, USA) software.

## Results

**Fig. 2:**
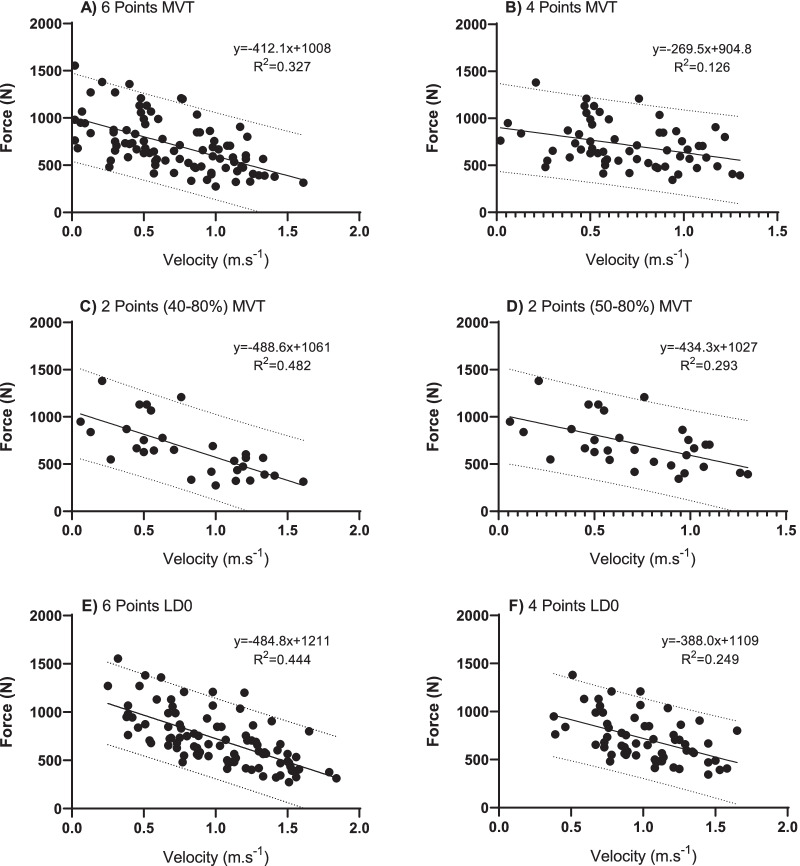

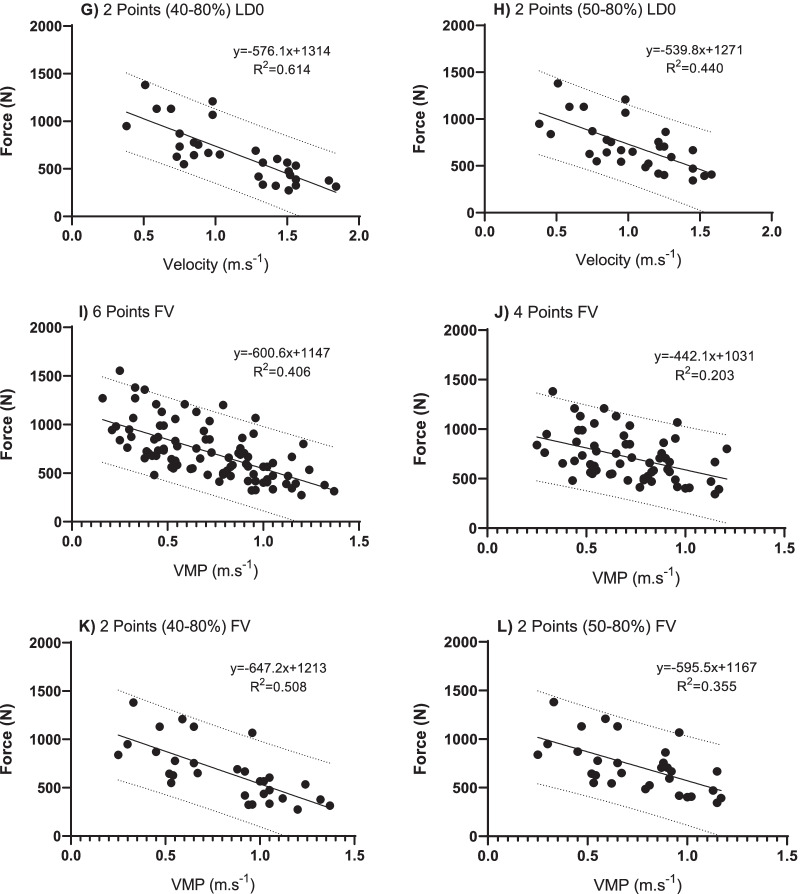
Linear Regression Model through the Force–Velocity relation between bar velocity and different percentages of 1 repetition maximum (1RM) in the Adapted Bench Press with free weight, in the minimum velocity limit (MVT), load at zero velocity (LD0) and velocity of force (FV). In each column are the linear regression line and the prediction equation based on 6 points (40, 50, 60, 70 80 and 90%), 4 points (50, 60, 70, 80%), 2 points (40–80%) and 2 points (50–80%)

Figure 2 shows the relationships between the bar velocities and different percentages of 1 repetition maximum (1RM) in the Adapted Bench Press with free weight. For MVT, LD0, and FV, the highest coefficient observed was 2 points (40–80%).

**Fig. 3:**
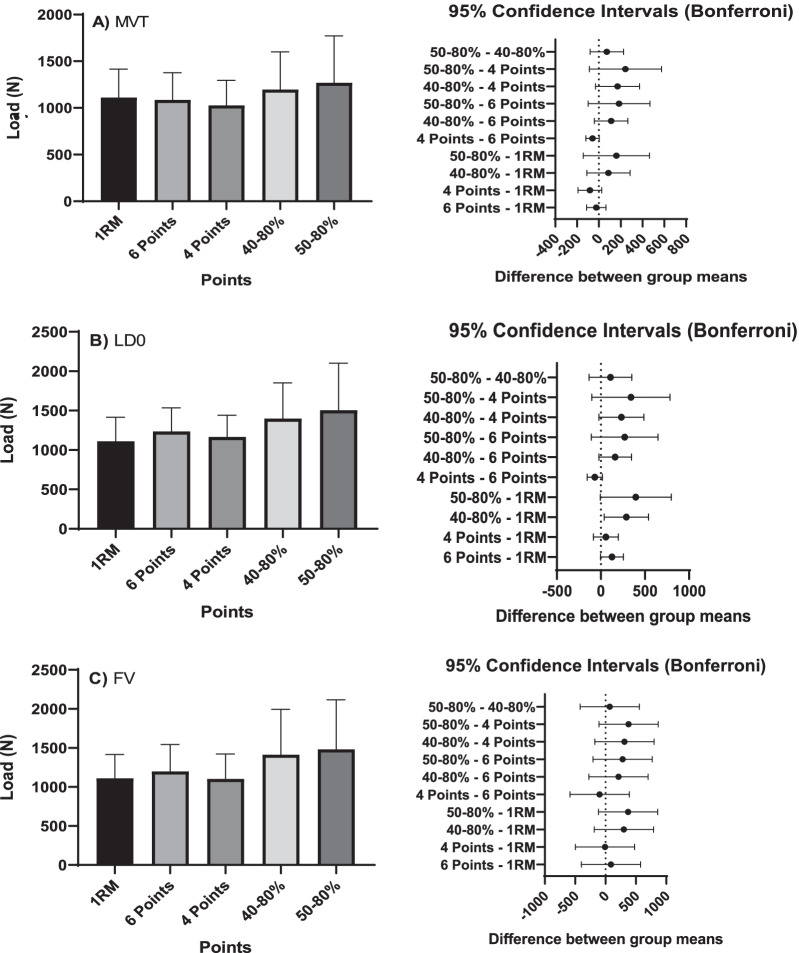
Results of predicted values at 6, 4, 40–80% and 50% using the Minimum Velocity Limit (MVT), Load at Zero Velocity (LD0) and velocity of force (FV) methods in relation to the measured value of 1RM and confidence interval of the test. No significant differences were found in any of the methods in relation to the 1RM assessed (*p* < 0.05)

Figure 3 shows the evaluations of predicted and measured results for 1RM (ANOVA) in 6, 4, 40–80% and 50%, in MVT, LD0, and FV. Regardless of the method, the highest load was observed between 50–80%, but without significant differences for the other loads.

Table 1 shows the intra-class correlation coefficient and the variation coefficient for the bench press test, adapted with 1RM (N) free weight, as predicted from the load-velocity relationship.

**Table 1:** Mean, standard deviation, Intraclass correlation coefficient and variation coefficient in the Force–velocity (F–V) parameters obtained from multiple load methods and two loads selected relating to 1RM in the Adapted Bench Press

Results	1RM (N) (X ± DP)	ICC (95% CI)	CV (95% CI)
1 RM	1108.53 ± 307.10		
*MVT (1RM predict)*			
6 Points Method MVT	1084.01 ± 294.04	0.97 (0.91–0.99)	0.6% (0.4–0.7)
4 Points Method MVT	1025.14 ± 270.19	0.93 (0.74–0.98)	1.4% (1.0–1.7)
Two-load (40–80%) MVT	1195.86 ± 404.70	0.88 (0.64–0.94)	6.1% (4.2–8.0)
Two-load (50–80%) MVT	1268.15 ± 502.01	0.76 (0.31–0.92)	12.5% (8.3–16.7)
*LD0 (1RM predict)*			
6 Points Method LD0	1234.37 ± 301.00	0.90 (0.49–0.97)	3.3% (2.4–4.2)
4 Points Method LD0	1164.05 ± 276.59	0.91 (0.74–0.97)	3.9% (2.9–4.9)
Two-load (40–80%) LD0	1396.22 ± 454.95	0.77 (0.47–0.92)	4.9% (3.4–6.4)
Two-load (50–80%) LD0	1504.12 ± 597.34	0.63 (0.17–0.86)	12.0% (7.9–16.1)
*FV (1RM predict)*			
6 Points Method FV	1197.66 ± 345.82	0.95 (0.76–0.99)	1.2% (0.8–1.5)
4 Points Method FV	1100.07 ± 320.94	0.97 (0.90–0.99)	1.5% (1.1–1.9)
Two-load (40–80%) FV	1410.83 ± 584.67	0.67 (0.04–0.89)	13.7% (2.0–27.0)
Two-load (50–80%) FV	1479.20 ± 635.95	0.60 (0.10–0.86)	15.3% (9.8–20.8)

*1RM* one repetition maximum, *X* ± *DP* mean ± standard deviation, *ICC* intraclass correlation, *CV* coefficient of variation

**Fig. 4:**
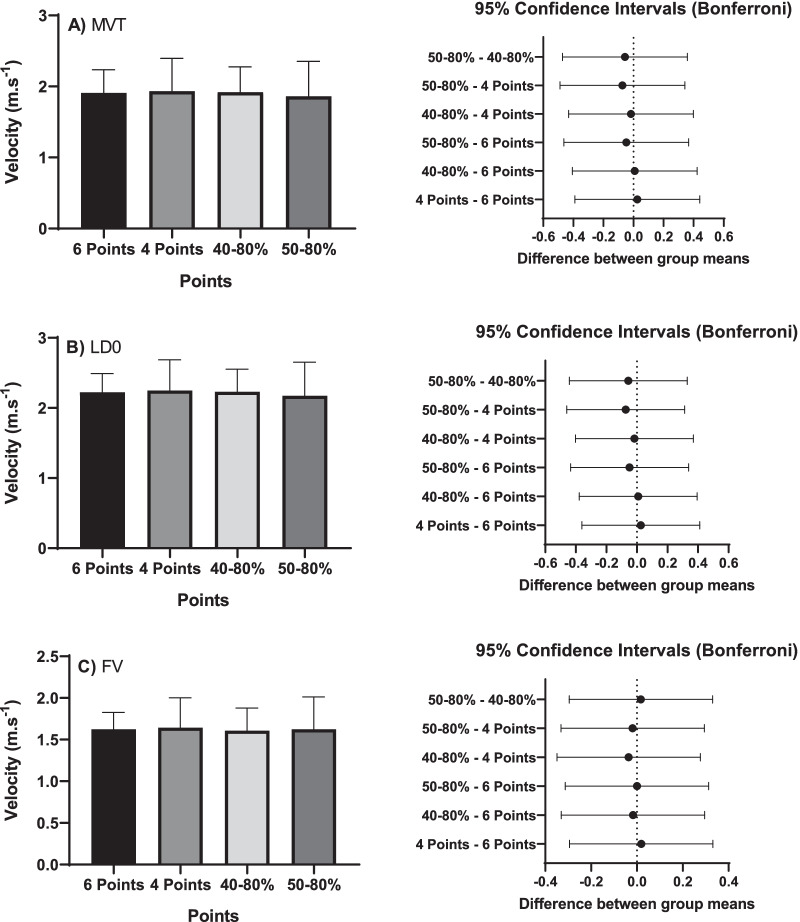
Results for predicted velocity values (ms-1) at 6, 4, 40–80% and 50% using the Minimum Velocity Limit (MVT), Load at Zero Velocity (LD0) and velocity of force (FV) in relation to the predicted values and confidence interval of the test

In Fig. 4, the evaluations of the results predicted and measured for velocity (ANOVA) are found in 6, 4, 40–80%, and 50%, in the MVT, LD0, and FV methods. No significant differences were found in any of the methods in relation to the points used in the evaluation (*p* < 0.05).

Table 2 shows the intra-class correlation coefficient and the variation coefficient for the bench press test, adapted with the free weight of the estimated velocity (m.s^−1^), as predicted from the load-velocity relationship.

**Table 2:** Intraclass correlation coefficient and variation coefficient in the Force–Velocity (F–V) parameters obtained from multiple load methods and two loads selected in relation to Velocity in 6 Points in the Adapted Bench Press

Results	Velocity (m.s^–1^) (X ± SD)	ICC (95% CI)	CV (95% CI)
*MVT (velocity predict)*			
6 Points Method MVT	1.91 ± 0.33		
4 Points Method MVT	1.93 ± 0.46	0.81 (0.40–0.93)	6.7% (5.3–8.1)
Two-load (40–80%) MVT	1.92 ± 0.36	0.94 (0.81–0.98)	1.5% (1.2–1.7)
Two-load (50–80%) MVT	1.86 ± 0.49	0.81 (0.43–0.94)	9.3% (7.2–11.3)
*LD0 (velocity predict)*			
6 Points Method LD0	2.22 ± 0.29		
4 Points Method LD0	2.25 ± 0.44	0.75 (0.23–0.92)	7.4% (6.2–8.6)
Two-load (40–80%) LD0	2.23 ± 0.32	0.91 (0.74–0.97)	2.4% (2.1–2.7)
Two-load (50–80%) LD0	2.18 ± 0.48	0.77 (0.32–0.92)	9.9% (8.2–11.6)
*FV (velocity predict)*			
6 Points Method FV	1.62 ± 0.20		
4 Points Method FV	1.64 ± 0.36	0.77 (0.30–0.92)	9.4% (7.8–11.1)
Two-load (40–80%) FV	1.61 ± 0.27	0.91 (0.73–0.97)	4.5% (3.9–5.2)
Two-load (50–80%) FV	1.62 ± 0.39	0.75 (0.22–0.92)	11.5% (9.3–13.6)

**Fig. 5:**
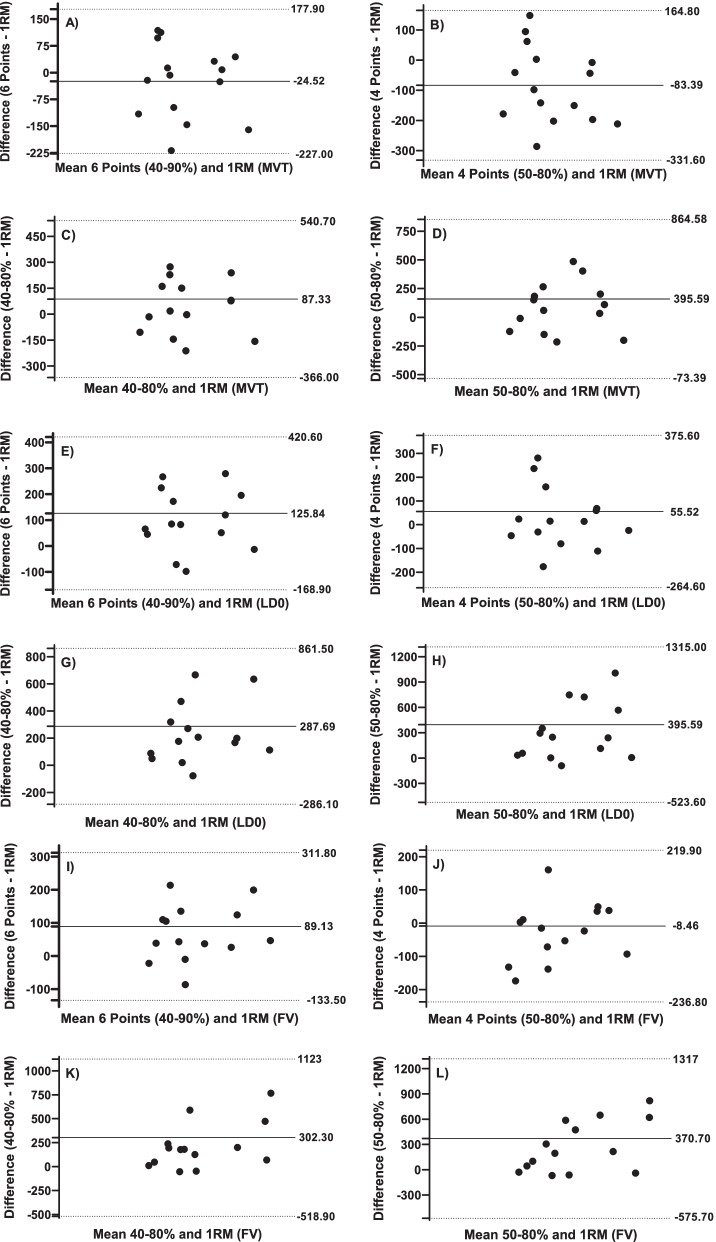
Bland–Altman plots showing differences between the parameters obtained from the 6-point and 4-point load method, and two points (40–80% and 50–80%) in the minimum velocity Limit (MVT), Load at zero velocity (LD0) and velocity of force (FV). Each graph represents the mean difference and 95% limits of agreement (dashed lines), along with the regression line (solid line)

In Fig. 5, the Bland–Altman Charts for MVT, LD0, and FV for the 6-point and 4-point load methods, and the two points (40–80% and 50–80%) are shown.

## Discussion

The study aimed to verify the highest precision of the multi-point method using 6 points (loads 40, 50, 60, 70, 80, and 90% of 1RM), 4 points (50, 60, 70, and 80% of 1RM) and two 2 points (40–80% and 50–80% of 1RM), through the MVT, LD0, and FV methods in Paralympic Powerlifting using free weights. The current results showed that the multiple (4 and 6) points method provides a good results of the F–V relationship with a good precision, and that the MVT, LD0, and FV methods present reliable values in the bench press for disabled people and national-level Paralympic Powerlifting athletes. The Force–velocity relationship in the MVT method for 6 and 4 points was R^2^ = 0.327 and 0.126, respectively, in the LD0 for 6 and 4 points, R^2^ = 0.444 and 0.249, respectively, and in the FV methods for 6 and 4 points R^2^ = 0.406 and 0.203, respectively. If an acceptable R^2^ with 0.75 (substantial), 0.50 (moderate), and 0.25 (weak) was adopted, values lower than 0.25 would be considered weak. Therefore, the 6-point method presented an acceptable coefficient of determination whilst the 4-point method presented a weak coefficient of determination. If a qualitative interpretation of the r coefficients defined by Hopkins (23) was made, the cutoff points would be 0–0.09 = trivial; 0.1–0.29 = small; 0.3–0.49 = moderate; 0.5–0.69 = large; 0.7–0.89 = very large; 0.9–0.99 = almost perfect; and 1 = perfect. Significant correlations were found in all methods. Moreover, the results of the 6 and 4 points methods showed an almost perfect relationship. The magnitude of the CV was based on the following parameters: poor (> 0.10%), moderate (5–10%), and good (5%) (12). In the same way, each one attends where the MVT presented 0.6 and 1.4, LD0 3.3 and 3.9 and FV 1.2 and 1.5, for 6 and 4 points respectively. Thus, it seems that for 6 points referring to 1RM, the results tend to be better than those with 4 points, for all methods.

The current findings are in accordance with previous research that showed that the F–V relationship is shown to be highly reliable and at least moderately valid, especially when assessed with variables using standard tests [52]. Nevertheless, other studies have shown that the F–V relationship may be inconsistent, and the discrepancy in results has demonstrated concurrent validity of F0 from moderate to high [53], and others have shown low or even insignificant results when related to the force measured directly. Low reliability was demonstrated through the MVT method with exercises with free weight for well-trained individuals (CV = 22.5%) [40]. However, in line with the present study, when evaluating the 1RM predicted through the MVT, although strongly correlated with the measured 1RM, the predicted 1RM scores overestimated the measured 1RM, which also occurred in the results of the multiple-point methods with 6 and 4 points [40]. This was attributed to the low reliability of the MVT variable, suggesting that the 1RM predicted by the MVT does not tend to be accurate to predict 1RM, which in part differs from the results of the present study.

The same prediction using the LD0, instead of the MVT [41, 52], was greater than 1RM, as in the results of the current study. Additionally, using the LD0 tends to overestimate the predicted 1RM [39], as also demonstrated through our resultsIn addition, researchers have used the Smith machine to predict 1RM for free weight exercises, which tends to be more technically demanding than exercise with machines, and therefore could present kinematic differences [41] that can explain the possible differences between what is predicted and the true 1RM.

However, within the FV estimation method [22], this study was not valid for free-weight exercise, contrary to the results of this study. In the present study, all participants performed their training with free weights only, which may be a justification for these results [54]. This study was performed with free weights, so with a velocity of 90% of 1RM and using the 6-point methods, it tends to induce technical differences between repetitions due to the high load [41]. This has been reported where variations with loads greater than 80% of 1 RM have been used [41]. In addition to the above, in Paralympic Powerlifting, the legs must be extended over the bench and the transfer of strength tends to be reduced with the adapted bench press, which can bring difficulty in stabilization and impact the the movement [36].

In the current study we found that the two-point method can be used with accuracy, using the same criteria for the 40–80% and 50–80% methods. The results show that in the two-point method, the R2 was 0.482 and 0.293 in the MVT method, being 0.614 and 0.440 with the LD0 as predictor, and 0.508 and 0.355 with the FV as predictor, (40–80% and 50–80%, respectively). The ICC presented in MVT was 0.88 and 0.76, in LD0 0.77 and 0.63, and in FV it was 0.67 and 0.60. The CV in MVT was 6.1 and 12.5, the LD0 4.9 and 12.0, and the FV 13.7 and 15.3, for 40–80% and 50–80%, respectively. There was also an overestimation of the 1RM predicted in all methods. Our findings were supported by the assumptions that the F–V has an independent relationship at zero values and with a reliable and moderately valid linear relationship [18, 23]. The F–V relationship tends to individualize different levels of physical fitness [55] and different modalities [56]. In other words, the idea is to use two-point methods to explain differences between participants in ballistic deficits, providing an individualized intervention [57]. Thus, the strong and approximately linear relation to F–V has been used to predict % 1RM [33], emphasizing that the load-velocity relationship tends to be specific for each exercise [33]. The linearity of the force–velocity and load-velocity relationships tend to allow a viable prediction to be obtained through two loads [47, 52] using a linear regression modelling [17, 58].

It is likely that the two-point method is more time-efficient, as it can be performed in conjunction with the warm-up and is even suitable for assessing injuries, as differences were observed between injured and uninjured players [21]. Some limitations have been addressed [57], with an emphasis on the specificity of the tests [59]. This study focused on Paralympic Powerlifting athletes, aiming to provide more tools for this segment. The determination of the points does not usually occur randomly. In this study, the rules of the sport were taken into account, in which the bar weighs 20 kg and the competition rulebook requires the placement of fastening clips, amounting to a minimum weight of 25 kg on the bar. So, for an athlete who lifts 80 KG in 1 RM, 30% of 1RM would be 24 kg,; so the research started with 40% of 1RM. One study evaluated the effect of distance between experimental points on the reliability and validity of the method [47]. The individuals were evaluated in the bench press exercise with loads of 20–70% 1RM, 30–60% 1RM, and 40–50% 1RM. The authors concluded that there was a decrease in the reliability and validity of the F–V parameters with the proximity of the points 40–50% 1RM (coefficient of variation [CV] = 18.0%; r = 0.64), 30–60% 1RM (CV = 7.3%; r = 0.94), 20–70% of 1RM (CV = 5.5%; r = 0.98). In the evaluation, it was determined that there should be a point closer to the maximum velocity with zero load, and another one closer to the maximum force with zero velocity.

Theoretically, from a physiological point of view, the F–V relation would be almost linear in values greater than 40% [60], and best represented by a linear function at forces greater than 40% (*R*^2^ = 0,996) while following a curvilinear function below that level [61]. On the other hand, it has already been mentioned that in loads of 90 and 100%, technical differences tend to be induced between repetitions due to the high load, and this has been reported with loads greater than 80% of 1 RM [41]. Confirming this, it has already been reported that the loads tend to be linear close to 80% and, above these values, it is likely not to preserve linearity [62]. Hence, the more stable loads should be between 40 and 80% of 1RM [41, 58]. Other authors suggest a range of 30 to 80% of 1RM [39], considering the number of attempts to avoid muscle fatigue (18). Finally, the last load should be close to 80% of 1RM [39]. Thus, the results indicate that the two-point loads of 40 and 80% were the ones that seemed to have the greatest relationship in the force and velocity indicators, mainly when MVT and LD0 wwre used as predictors.

The use of free weights presented good reliability in the bench press adapted for disabled people and national-level Paralympic Powerlifting athletes. The Force–Velocity relationship in the Bench Press has been reported in previous studies using the Smith machine [14], and also in the free bench press [41]. The use of the free bench press tends to have a greater transfer than in the Smith machine [14]. These results are in agreement with some previous studies [13, 16, 39] that have already described the very close linear relationship that exists between force and velocity, where athletes' experience can interfere with strength and conditioning [13]. Regarding the validity of the 1RM predictions based on velocity, previous research has already shown that the MVT and LD0 measures showed very strong correlations with the 1RM measured in the bench press (r = 0.95–0.98 and r = 0.99, respectively) [13, 16, 39]. The results of this study agree with other studies [40, 41], as both MVT and LD0 had better 1 RM predictions for all points, while the same did not occur with the FV measure.

Regarding PP specifically, a study conducted by Loturco et al. [16] showed that the load-velocity relationship was strong and accurate for Paralympic powerlifters, especially at higher load intensities (≥ 70% 1RM). They also mentioned that the 1RM tests were performed at lower velocities than those previously reported in the literature. This study found no such results and the velocity of the Powerlifters was close to that of non-disabled people or even above (VMP = 0.17 ± 0.1 and VMax 0.32 ± 0.1 m.s^–1^). On the other hand [58], it was observed that posture and transfer of strength interfered with the performance in the bench press. Indeed, it was observed that in the study by Loturco et al. [16], the sample was small (eight men, five women, and four dwarfs), the calculation of the sample size was not provided, and the force of 1RM variation was large (standard deviation of up to 30% in the measured force and 41% in the predicted). Thus, there was a greater possibility of type II error in that study. A small number of participants can cause a violation of the assumption of the normality of the variables [63], where the p-value for small samples can be mistaken [48]. In addition, considering that in PP the legs must be extended on the bench, there is a tendency to reduce the transfer of strength in the bench press, where there would be difficulty in maintaining force and velocity [36].

The Bland–Altman analysis demonstrated great overestimations and underestimations for different participants. Despite significant statistical trends, it must be emphasized that very large samples are needed to provide conclusive information on trends through Bland–Altman analysis [49]. Thus, further studies are required, since estimates of 1RM based on load-velocity relationships may not be accurate enough to determine the true load. It seems that the multiple point methods (4 and 6 points) using the MVT, LD0, and FV as predictors, showed statistical relevance for the prediction of 1RM. The two-point method (40–80%), also showed statistical significance, while the same did not occur with higher loads in this same model (50–80%).

### Practical applications

The methods herein proposed, mainly those with multiple points (4 and 6 points) and with two points (40–80%) are a possibility for monitoring the training load in PP, and these can be evaluated every day during warm-up. It is recommended that coaches use the best predictive methods of F–V as a control tool throughout the periodization to monitor athlete performance. The three methods, MVT, LD0, and FV, showed the same predictive power, so coaches should choose the one that athletes feel most comfortable with. Furthermore, the velocity measurement in the study was performed using a linear encoder, which has affordable prices and can even be performed with validated statistical programs.

## Data Availability

The data presented in this study are available on request from the corresponding author.

## Abbreviations

MVT: Minimum velocity limit
LD0: Load at zero velocity
FV: Velocity of force
PP: Paralympic powerlifting
1RM: One repetition maximum
F–V: Relation to force–velocity
F0: Maximum production of force
V0: The maximum velocity
Pmax: Power
N: Newtons
